# N-acetyl-seryl-aspartyl-lysyl-proline Inhibits Diabetes-Associated Kidney Fibrosis and Endothelial-Mesenchymal Transition

**DOI:** 10.1155/2014/696475

**Published:** 2014-03-24

**Authors:** Takako Nagai, Megumi Kanasaki, Swayam Prakash Srivastava, Yuka Nakamura, Yasuhito Ishigaki, Munehiro Kitada, Sen Shi, Keizo Kanasaki, Daisuke Koya

**Affiliations:** ^1^Department of Diabetology and Endocrinology, Kanazawa Medical University, Uchinada, Ishikawa 920-0293, Japan; ^2^Medical Research Institute, Kanazawa Medical University, Uchinada, Ishikawa 920-0293, Japan

## Abstract

Endothelial-to-mesenchymal transition (EndMT) emerges as an important source of fibroblasts. MicroRNA let-7 exhibits anti-EndMT effects and fibroblast growth factor (FGF) receptor has been shown to be an important in microRNA let-7 expression. The endogenous antifibrotic peptide N-acetyl-seryl-aspartyl-lysyl-proline (AcSDKP) is a substrate of angiotensin-converting enzyme (ACE). Here, we found that AcSDKP inhibited the EndMT and exhibited fibrotic effects that were associated with FGF receptor-mediated anti-fibrotic program. Conventional ACE inhibitor plus AcSDKP ameliorated kidney fibrosis and inhibited EndMT compared to therapy with the ACE inhibitor alone in diabetic CD-1 mice. The endogenous AcSDKP levels were suppressed in diabetic animals. Cytokines induced cultured endothelial cells into EndMT; coincubation with AcSDKP inhibited EndMT. Expression of microRNA let-7 family was suppressed in the diabetic kidney; antifibrotic and anti-EndMT effects of AcSDKP were associated with the restoration of microRNA let-7 levels. AcSDKP restored diabetes- or cytokines-suppressed FGF receptor expression/phosphorylation into normal levels both in vivo and in vitro. These results suggest that AcSDKP is an endogenous antifibrotic molecule that has the potential to cure diabetic kidney fibrosis via an inhibition of the EndMT associated with the restoration of FGF receptor and microRNA let-7.

## 1. Introduction

Diabetic nephropathy is leading course of end-stage kidney disease and kidney fibrosis is the final common pathway in progressive kidney diseases. The fibroblasts that play a role in kidney fibrosis are believed to be heterogeneous [1]. Recently, the endothelial-to-mesenchymal transition (EndMT) has emerged as an important source of myofibroblasts or activated fibroblasts [2].

N-acetyl-seryl-aspartyl-lysyl-proline (AcSDKP) is a tetrapeptide that is normally present in human plasma and is hydrolyzed by angiotensin-converting enzyme (ACE); ACE-inhibitor (ACE-I) treatment increases the plasma level of AcSDKP by fivefold [3]. We demonstrated that AcSDKP has an antifibrotic activity; that is, AcSDKP inhibits the transforming growth factor (TGF)-*β*-induced fibrogenic gene expression in human mesangial cells by inhibiting the smad 2/3 signaling [4] and rescues glomerular damage in db/db mice [5]. AcSDKP reportedly exhibits antifibrotic and organ protective effects in various experimental models [6–14].

We aimed to investigate whether antifibrotic peptide AcSDKP exerts additive antifibrotic effects associated with the inhibition of EndMT on top of the conventional ACE-I based therapy in fibrotic kidney model of diabetic mice.

## 2. Materials and Methods

### 2.1. Reagents and Antibodies

The AcSDKP was a gift from Dr. Omata from Asabio Bio Technology (Osaka, Japan). Imidapril (ACE-I) and TA-606 (ARB) were provided by Mitsubishi Tanabe Pharma (Osaka Japan) through an MTA agreement. The mouse monoclonal anti-human CD31 antibody was purchased from R&D Systems (Minneapolis, MN, USA), and the rat polyclonal anti-mouse CD31 antibody was purchased from EMFRET Analytics GmbH & Co. KG (Eibelstadt, Germany). The polyclonal rabbit anti-*α*SMA antibody was obtained from Gene Tex (Irvine, CA, USA). The rabbit polyclonal anti-SM22*α* antibody and monoclonal anti-VE-cadherin antibody were obtained from Novus Biological (Littleton, CO, USA). The polyclonal anti-GAPDH and anti-TGF-*β*-receptor I antibodies were obtained from Sigma-Aldrich (St. Louis, MO, USA). Fluorescein-, rhodamine-, and Alexa 647-conjugated secondary antibodies were obtained from Jackson ImmunoResearch (West Grove, PA, USA). Antifibroblast growth factor (FGF) receptor, anti-phospho-FGF receptor, and the HRP-conjugated secondary antibodies for Western blot detection were purchased from Cell Signaling Technology (Danvers, MA, USA). TGF-*β*2, tumor necrosis factor (TNF)-*α*, and interleukin (IL)-1*β* were purchased from PeproTech (Rocky Hill, NJ, USA).

### 2.2. Animal Experiments

We utilized a fibrotic diabetic kidney disease model, that is, streptozotocin- (STZ-) treated CD-1 mice [15]. Eight-week-old male CD-1 mice were obtained from Sankyo Lab Service (Tokyo, Japan). A single intraperitoneal injection of streptozotocin (STZ) (200 mg/kg) was given to the mice. We confirmed the induction of diabetes by a blood glucose level >16 mM at 2 weeks after the STZ injection. Sixteen weeks after the induction of diabetes, the diabetic mice were divided into the following four groups: (imidapril [2.5 mg/kg BW/day], AcSDKP [500 *μ*g/kg BW/day using an osmotic mini-pump], AcSDKP+imidapril, TA-606 [3 mg/kg BW/day], and nontreatment). Imidapril or TA-606 was provided in drinking water. All of the mice were euthanized 24 weeks after the induction of diabetes, and their blood pressure was monitored using the tail-cuff method with a BP-98A instrument (Softron Co. Beijing, China) within a week before euthanasia.

### 2.3. AcSDKP Measurements

Blood was harvested into a heparinized tube containing captopril (final concentration 10 *μ*mol/L) and centrifuged at 3,000 ×g for 15 min at 4°C. We obtained estimated plasma and urine Ac-SDKP concentrations using a competitive enzyme immunoassay kit (SPI-BIO, Massy, France) according to the manufacturer's instruction. Urine AcSDKP was normalized at the urine creatinine level.

### 2.4. EndMT Detection In Vivo

EndMT were determined by double-labeling with antibodies against CD31 and *α*SMA, or with antibodies against CD31 and FSP1 on frozen sections (5-*μ*m). The immunolabeled sections were analyzed using fluorescence microscopy (Axio Vert.A1, Carl Zeiss Microscopy GmbH, Jena, Germany). We obtained images of six different fields of view at 300x magnification and performed quantification. All immunolabelings were analyzed with appropriate negative control, including isotype IgG.

### 2.5. Morphological Evaluation

We determined the surface area of 10 glomeruli in each mouse using ImageJ software. A point-counting method was utilized to evaluate the relative area of the mesangial matrix (%). We analyzed 10 PAS-stained glomeruli from each mouse using a digital microscope screen grid containing 540 (27 × 20) points and employing Adobe Photoshop Element 6.0. The number of grid points on the mesangial tissue was divided by the total number of points in the glomerulus to obtain the mesangial area in a given glomerulus as the percentage of the total area of the glomerulus. Images of Masson's trichrome-stained tissue were analyzed using ImageJ software, and the fibrotic areas were quantified. For each mouse, images of six different fields of view at 100x magnification were evaluated.

### 2.6. In Vitro EndMT

Human umbilical vein endothelial cells (HUVEC) (Kurabo Industries Ltd., Osaka, Japan) cultured in HuMedia-EG2 medium and human dermal microvascular endothelial cells (HMVEC) (Lonza, Basel, Switzerland) cultured in EGM medium were used for the experiments. When cells grown on an adhesion reagent (Kurabo Medical, Osaka, Japan) reached 70% confluence, a combination of TGF-*β*2 (2.5 ng/mL), TNF-*α* (1.0 ng/mL), and IL-1*β* (2.0 ng/mL) was added to the experimental medium (a mixture of Humedia-EG2 in serum-free RPMI, 1 : 3 ratio) for an indicated interval, with or without a 2 h preincubation in AcSDKP (100 nM).

### 2.7. Western Blotting

Protein lysates were denatured in a SDS sample buffer at 100°C for 5 min. After centrifugation (17,000 ×g for 10 min at 4°C), supernatants were separated on SDS-polyacrylamide gels and blotted onto PVDF membranes (Pall Corporation, Pensacola, FL, USA) using the semidry method. The immunoreactive bands were developed using an enhanced chemiluminescence (ECL) detection system (Pierce Biotechnology, Rockford, IL, USA) and detected using an ImageQuant LAS 400 digital biomolecular imaging system (GE Healthcare Life Sciences, Uppsala, Sweden).

### 2.8. MicroRNA Array Analysis

Total RNA was isolated using a miRNeasy kit (Qiagen). After dephosphorylation and denaturation, the total RNA was labeled with cyanine 3-pCp and subsequently hybridized to an Agilent mouse microRNA microarray (release version 15) using the microRNA Complete Labeling and Hyb Kit (Agilent Technologies, Inc.). After hybridization for 20 h, the slides were washed using the Gene Expression Wash Buffer (Agilent Technologies, Inc.), scanned using an Agilent Scanner G2565BA, and processed and analyzed using Agilent Feature Extraction Software version 9.5.1. The raw data were analyzed using GeneSpring GX software version 12.5 (Agilent Technologies, Inc.).

### 2.9. MicroRNA Isolation and qPCR

The kidney tissues that had been maintained at −80°C were first incubated in RNA*later* ^*R*^-ICE (Life Technologies) for 16 h at −20°C before homogenization. The tissues were homogenized on ice and the microRNA was extracted. Total cDNA was synthesized using a miScript II RT kit (Qiagen) and the real-time quantification of microRNA expression was performed using a miScript SYBR Green PCR kit (Qiagen). Samples of 3 ng of cDNA were used in the qPCR experiment. The primers for Mm_let-7f-1, Mm_let-7 g-1, and Mm_let-7i-1 were from the miScript Primer Assay designed by Qiagen. The mature microRNA sequences were 5′CUAUACAAUCUAUUGCCUUCCC for Mm_let-7f-1, 5′ACUGUACAGGCCACUGCCUUGC for Mm_let-7 g-1, and 5′CUGCGCAAGCUACUGCCUUGCU for Mm_let-7i-1. All of the experiments were performed in triplicate, and Hs_RNU6-2_1 (Qiagen) was used as an endogenous control for normalization.

### 2.10. Statistical Analysis

The data are expressed as the mean ± SEM values. The Mann-Whitney* U*-test was used to determine the significance. Statistical significance was defined as *P* < 0.05. GraphPad Prism software (ver. 5.0f) was used for the statistical analyses.

## 3. Results

### 3.1. Antifibrotic Effect of AcSDKP on the Top of ACE-I

**Figure 1:**
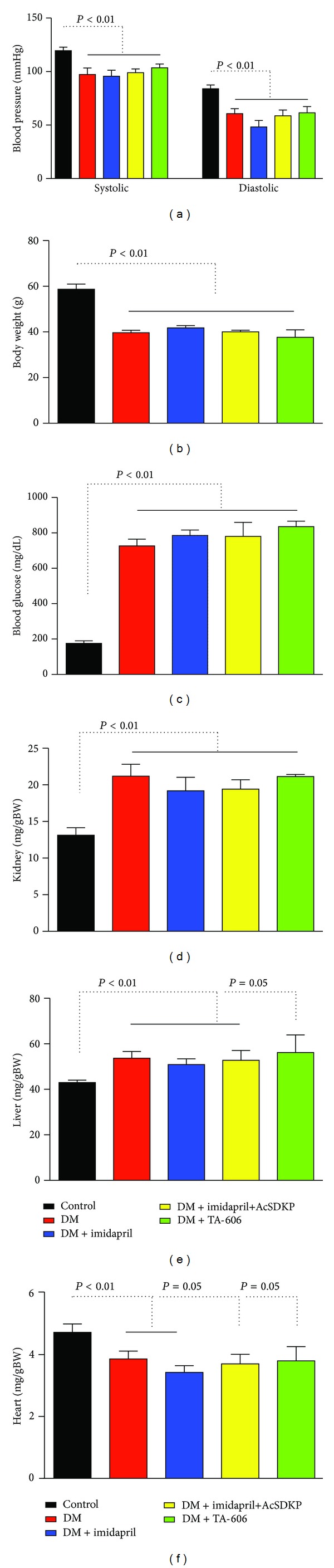
Characteristics of the experimental animals. (a) Blood pressure was measured within 1 week before the mice were euthanized. (b) Body weight. (c) Blood glucose level at the time of euthanasia. (d–f) Organ weights. Kidney (d), liver (e), and heart (f) weights relative to body weight (g) are shown. The data are expressed as the mean ± SEM values. Control: *n* = 7, STZ-induced diabetes: *n* = 5, diabetes treated with imidapril: *n* = 6, diabetes treated with imidapril+AcSDKP: *n* = 5, diabetes treated with TA-606: *n* = 3. Diabetic mice are designated as DM.

The characteristics of the mice in each group are shown in Figure 1. Compared to the control mice, the diabetic mice had lower blood pressure, weighed less, and had higher blood glucose; their kidneys and livers weighed more, and their hearts weighed less. Treatment with imidapril, imidapril+AcSDKP, or the angiotensin II receptor blocker (ARB) TA-606 [16] did not alter the blood pressure, body weight, or organ weights of the diabetic mice (Figure 1).

**Figure 2:**
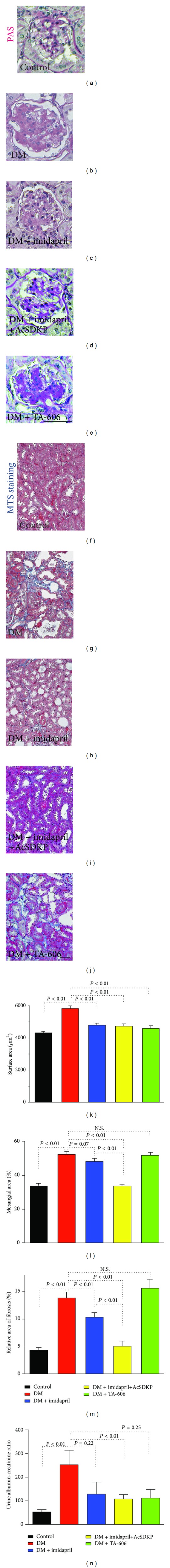
AcSDKP exerts antifibrotic effects on diabetic kidney disease. ((a)–(e)) Representative periodic acid Schiff- (PAS-) stained kidney samples from the indicated groups of mice. The original magnification of the images was 300x. ((f)–(j)) Representative images of Masson's trichrome-stained (MTS) samples from the indicated groups of mice are shown. The original magnification of the images was 200x. ((k)–(m)) Morphometric analysis. The glomerular surface area (k), relative mesangial area (l), and relative area of fibrosis (m) were analyzed by the method described in the Methods section. (n) Urinary albumin/creatinine ratio. The data are expressed as the mean ± SEM values. Control: *n* = 7, STZ-induced diabetes: *n* = 5, diabetes treated with imidapril: *n* = 6, diabetes treated with imidapril+AcSDKP: *n* = 5, diabetes treated with TA-606: *n* = 3. Diabetic mice are designated as DM. Scale bar: 50 *μ*m.

The diabetic CD-1 mice exhibited glomerulomegaly and the accumulation of a PAS-positive matrix in the glomeruli (Figures 2(a), 2(b), 2(k), and 2(l)). Imidapril, imidapril+AcSDKP, and TA-606 inhibited the expansion of the glomerular surface area (Figures 2(b), 2(c), 2(d), 2(e), and 2(k)). The expansion of the mesangial area was partially inhibited by treatment with imidapril, and imidapril+AcSDKP treatment nearly completely inhibited the matrix expansion (Figures 2(b), 2(c), 2(d), and 2(l)). However, TA-606 did not inhibit the expansion of the mesangial area in the diabetic kidney (Figures 2(b), 2(e), and 2(l)).

MTS staining revealed massive tubulointerstitial fibrosis in the diabetic mice that was not exhibited in the control mice (Figures 2(f), 2(g), and 2(m)). A comparison to the untreated diabetic mice showed that imidapril partially decreased interstitial fibrosis (Figures 2(g), 2(h), and 2(m)) and that imidapril+AcSDKP combination nearly completely inhibited the interstitial fibrosis (Figures 2(g), 2(i), and 2(m)). However, TA-606 treatment did not suppress kidney fibrosis (Figures 2(g), 2(h), 2(i), 2(j), and 2(m)). Compared to the control mice, the diabetic mice exhibited enhanced urinary albumin excretion (Figure 2(n)). Imidapril and TA-606 treatment inhibited the trend of increased urinary albumin excretion; imidapril+AcSDKP significantly inhibited urine albumin excretion in the diabetic mice (Figure 2(n)).

### 3.2. The Levels of AcSDKP and Kidney Fibrosis

**Figure 3:**
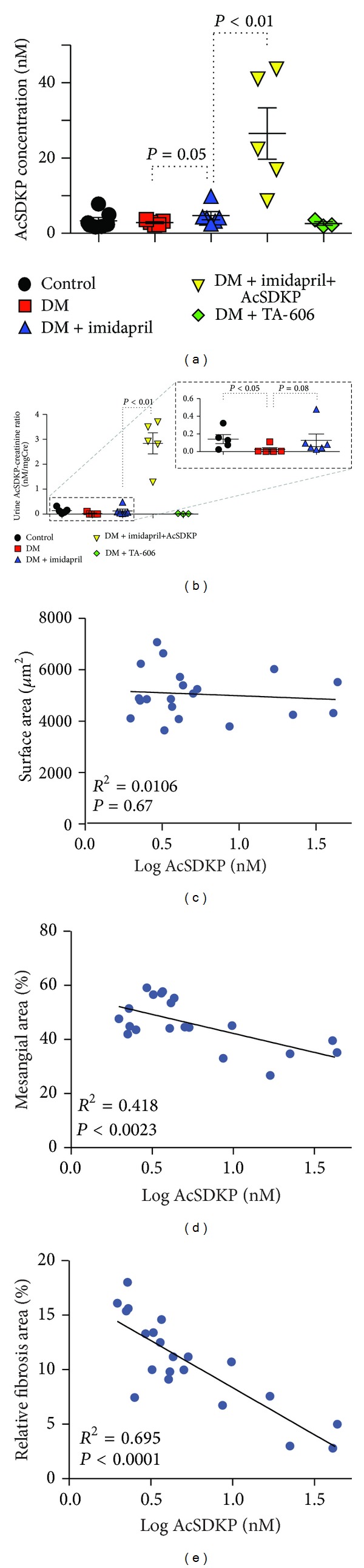
Negative correlation between the concentration of AcSDKP and mesangial area or relative fibrosis area. (a) Plasma concentration of AcSDKP in the indicated group of mice. (b) Urinary AcSDKP/creatinine ratio. Inset in (b) shows an enlargement of the dotted square area of the graph. The data are expressed as the mean ± SEM values. Diabetes is designated as DM. Control: *n* = 7 (urine, *n* = 5), STZ-induced diabetes: *n* = 5, diabetes treated with imidapril: *n* = 6, diabetes treated with imidapril+AcSDKP: *n* = 5, diabetes treated with TA-606: *n* = 3. ((c)–(e)) Linear regression analysis of the relationship between the plasma AcSDKP concentration and the values for the morphometric parameters. Glomerular surface area (c), relative mesangial area (d), and relative fibrosis area (e) are shown. The AcSDKP levels were plotted using a log conversion. Only diabetic animals were analyzed.

The plasma AcSDKP concentration demonstrated a decreased trend in the diabetic mice (Figure 3(a)). The AcSDKP concentration was high in the mice treated with imidapril (Figure 3(a): *P* = 0.05). When AcSDKP was added to the imidapril treatment, the concentration of AcSDKP was additionally increased by 5.5-fold (Figure 3(a)). The mice treated with AcSDKP alone exhibited several antifibrotic effects of diabetic mice and displayed higher plasma levels of AcSDKP compared to the diabetic mice, as shown in several fibrotic animal models (see Supplementary Figure  1 available online at http://dx.doi.org/10.1155/2014/696475) [6–14]. The ratio of the AcSDKP concentration to the creatinine concentration in urine exhibited a trend similar to that of the plasma AcSDKP levels, except that the urinary AcSDKP levels of diabetic mice were significantly lower than those of control mice (Figure 3(b)). TA-606 treatment in diabetic mice did not alter the level of AcSDKP either in plasma or in urine (Figures 3(a) and 3(b)). There was no correlation between the plasma AcSDKP concentration and the glomerular surface area in the diabetic mice (Figure 3(c)). In contrast, we found a negative correlation between the plasma concentration of AcSDKP and the mesangial area (Figure 3(d)) or the relative fibrotic area of the kidneys (Figure 3(e)).

### 3.3. AcSDKP Inhibited EndMT

**Figure 4:**
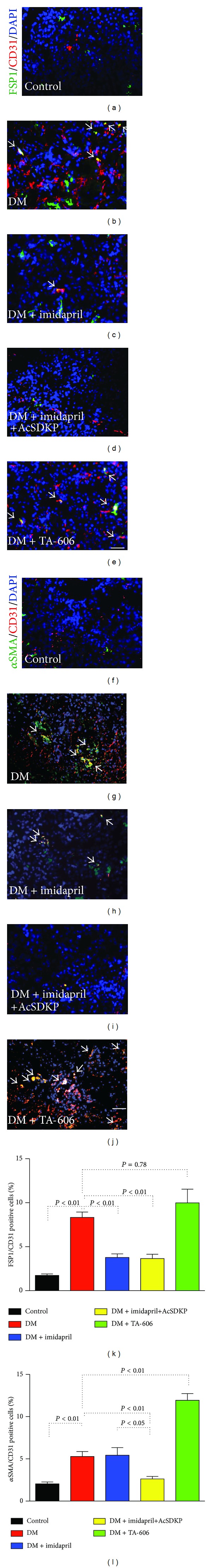
AcSDKP inhibits EndMT in the diabetic kidney. ((a)–(e)) Immunolabeling for FSP1 and CD31 in a kidney from each group of mice. Arrows indicate cells that are double-labeled for FSP1 and CD31. ((f)–(j)) Immunolabeling for *α*SMA and CD31 in a kidney from each group of mice. Arrows indicate cells that are double-labeled for *α*SMA and CD31. Merged images with DAPI-stained nuclei are shown. Scale bar: 25 *μ*m. ((k), (l)) Quantification of cells undergoing EndMT. FSP1 and CD31 double-labeled cells (k) and *α*SMA and CD31 double-labeled cells (l) in each visual field were imaged using fluorescence microscopy and quantified. The data are expressed as the mean ± SEM values. Diabetes is designated as DM. For all of the groups except the group that received TA-606 (*n* = 3), *n* = 5 mice were analyzed.

An analysis of cells undergoing EndMT, which were identified by double-labeling with FSP1 and CD31 antibodies [FSP1(+); CD31(+)] or with *α*SMA and CD31 antibodies [*α*SMA(+); CD31(+)], showed that the diabetic kidneys contained significantly more cells undergoing EndMT than did the control kidneys (Figures 4(a), 4(b), 4(f), 4(g), 4(k), and 4(l)). Imidapril treatment decreased the number of FSP1(+); CD31(+) cells but did not affect the number of *α*SMA(+); CD31(+) cells compared to the untreated diabetic mice (Figures 4(b), 4(c), 4(g), 4(h), 4(k), and 4(l)). Imidapril+AcSDKP combination therapy nearly completely inhibited the induction of cells undergoing EndMT (Figures 4(b), 4(d), 4(g), 4(i), 4(k), and 4(l)). However, TA-606 treatment did not reduce the number of FSP1(+); CD31(+) cells; furthermore, the number of *α*SMA(+); CD31(+) cells was increased by TA-606 treatment relative to the diabetic mice (Figures 4(b), 4(e), 4(g), 4(j), 4(k), and 4(l)).

**Figure 5:**
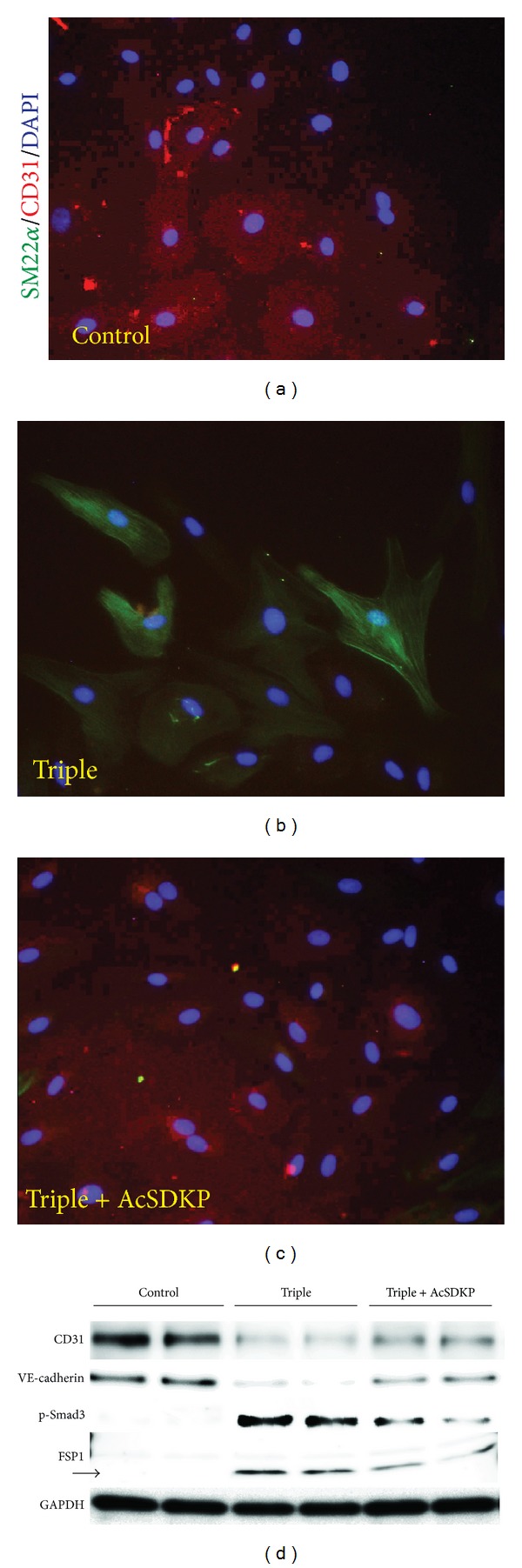
AcSDKP inhibits EndMT in vitro. HMVEC were exposed to a combination of cytokines (TGF-*β*2 at 2.5 ng/mL, IL-1*β* at 4 ng/mL, and TNF-*α* at 2 ng/mL) in the presence or absence of AcSDKP (100 nM) for 72 h. ((a)–(c)) Immunofluorescent microscopic analysis. The original magnification of the images was 200x. Merged images with DAPI-stained nuclei are shown. (d) Western blot analysis of the EndMT. HMVEC were stimulated with the triple cytokine mixture in the presence or absence of AcSDKP for 72 h. The cells were harvested, and the proteins were immunoblotted onto PVDF membranes. Chemiluminescence-detected bands were visualized. *n* = 2 in each group. GAPDH was used as the loading control.

When HMVEC were stimulated with a triple mixture of cytokines (TGF-*β*2, IL-1*β*, and TNF-*α*), the expression of the endothelial marker CD31 or VE-cadherin was suppressed; the expression of the mesenchymal marker FSP1 or SM22*α* was induced, suggesting induction of EndMT (Figure 5). AcSDKP preincubation inhibited cytokines-stimulated EndMT associated with the suppression of smad3 phosphorylation (Figure 5).

### 3.4. Endogenous Antifibrosis Program by AcSDKP through FGF Receptor

**Figure 6:**
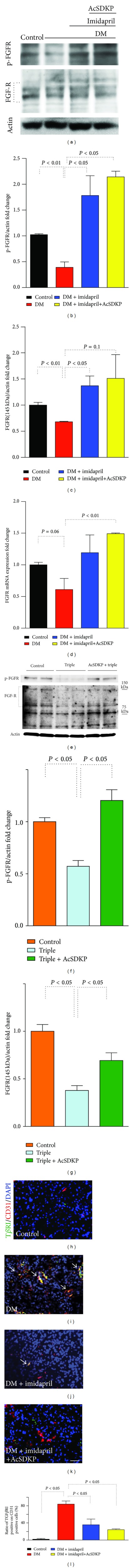
Antifibrotic effect of AcSDKP is associated with restoration of FGFR levels. (a) Western blot analysis of FGF receptor phosphorylation and total protein levels are shown. Actin is shown as a loading control. ((b)-(c)) Densitometric analysis of phospho-FGF receptor and total FGF receptor levels normalized to actin. The results are shown as the relative expression against the control animal values. The data are shown as the mean ± SEM values (*n* = 3). (d) qPCR analysis for FGF receptor in kidney. The data are shown as the mean ± SEM values. *n* = 3 in each group were analyzed. (e) Western blot analysis for FGF receptor levels and its phosphorylation. The same sample as Figure 5(d) was analyzed for FGF-R status. Representative results (*n* = 2) were shown. ((f), (g)) Densitometric analysis of phospho-FGF receptor and total FGF receptor levels normalized to actin. The results are shown as the relative expression against the control value. The data are shown as the mean ± SEM values (*n* = 4). TGF-*β* receptor I on endothelial cells in diabetic mice. ((h)–(k)). Immunolabeling for TGF-*β* receptor I (T*β*RI) and CD31 in a kidney from indicated group of mice. Arrows indicate cells that are double-labeled for T*β*RI and CD31. Scale bar: 25 *μ*m. (l) Quantification of cells expressing T*β*RI and CD31. Ratio of T*β*RI positive on all CD31 positive cells in each visual field among 4 images from one animal using fluorescence microscopy and quantified. The data are expressed as the mean ± SEM values. Diabetes is designated as DM. For all of the groups *n* = 4 mice were analyzed.

Chen et al. [19] reported that FGF receptor-mediated induction of microRNA let-7 family members, which exhibits kidney protective roles [17, 18], acts as negative regulators of the EndMT program via inhibition of the TGF-*β* signaling pathway [19]. FGF receptor phosphorylation and protein levels were suppressed in diabetic kidney [20]; imidapril treatment increased both the phosphorylation and protein levels of the FGF receptor in the diabetic kidney (Figures 6(a), 6(b), and 6(c)). A combination therapy exhibited stronger effects on the FGF receptor levels and phosphorylation (Figures 6(a), 6(b), and 6(c)). Such effects of intervention on the FGF receptor in diabetic mice were likely transcription-dependent (Figure 6(d)). When HMVEC was stimulated by mixture of cytokines, the protein levels and phosphorylation of the FGF receptor were significantly diminished; AcSDKP preincubation restored the FGF receptor levels (Figures 6(e), 6(f), and 6(g)).

Our microRNA array analysis of kidney samples revealed that expression of the microRNA mmu-let-7 family was suppressed in the diabetic kidney; we found that expression of most of the microRNA let-7 family members was restored by therapy with the combination of imidapril+AcSDKP (Supplementary Figure  2). qPCR analysis also confirmed that certain sets of microRNA let-7 were indeed inhibited in diabetic mice (Supplementary Figure  2); treatment with the imidapril+AcSDKP combination therapy completely restored their levels. The FGF receptor-microRNA let-7 family axis can suppress TGF-*β* receptor I levels [19, 21]. In agreement with this report, we observed that endothelial cells in diabetic mice exhibited strong expression of TGF-*β* receptor I; the combination therapy group nearly completely abolished such TGF-*β* receptor I expression on endothelial cells in the diabetic kidney (Figures 6(h), 6(i), 6(j), 6(k), and 6(l)).

## 4. Discussion

### 4.1. AcSDKP Inhibited EndMT in Fibrotic Kidney in Diabetes

EndMT has emerged as an important source of activated fibroblasts or myofibroblasts [1, 2, 22–24]. The EndMT has been shown to be associated with glomerulosclerosis in early diabetic kidney disease and tubulointerstitial fibrosis in a chronic type 1 diabetes kidney disease model [25]. The TGF-*β*-induced Smad signaling pathway plays an essential role in the EndMT [26]. In our analysis, AcSDKP inhibited both EndMT in diabetic kidney and in vitro culture cells associated with the inhibition of TGF-*β*/Smad signal transduction. It needs to be mentioned that we rather focused on EndMT in our analysis; there is a possibility that AcSDKP inhibits other fibroblast activation pathways, as reported elsewhere [27]. Also anti-inflammatory, antiapoptotic, and enhanced normal angiogenesis pathways would be contributed in the beneficial effects of AcSDKP [3].

### 4.2. Two Catalytic Sites of ACE and Endogenous Antifibrotic Program via AcSDKP

Mammalian ACE has two catalytic sites, the N- and C-terminal catalytic domains. These two catalytic domains may have different substrate selectivity. Although angiotensin-I can be converted to angiotensin-II by either catalytic domain, the C-terminal domain has a threefold higher affinity for angiotensin-I. Interestingly, only the N-terminal catalytic domain hydrolyzes AcSDKP [3]. Notably, each ACE-I exhibits a distinct affinity for one of the catalytic domains; for example, captopril exhibits a higher affinity for the N-terminal catalytic domain. Li et al. recently reported that mice deficient in the N-terminal catalytic domain of ACE exhibited an antifibrotic effect because of the accumulation of AcSDKP [28], which reveals the importance of the N-terminal domain for the antifibrotic activity of ACE-I [29–32].

### 4.3. AcSDKP Stimulates Antifibrotic Program

In our analysis, the concentration of AcSDKP was negatively associated with mesangial expansion and kidney fibrosis. Moreover, we found that endogenous AcSDKP levels were lower in the urine of diabetic animals with fibrotic kidneys. Similar to this observation, suppressed levels in other antifibrotic molecules, such as bone morphogenic protein 7 or its receptor-mediated signaling, in fibrotic kidney diseases have been reported elsewhere [33]. Apart from diabetic kidney disease model, the association between the levels of AcSDKP, other fibrotic kidney disease, and human kidney diseases needs further investigation. These results suggest that high intrarenal ACE activity in the diabetic kidney reduced the level of endogenous AcSDKP; this reduction of the antifibrotic peptide AcSDKP accelerated the fibrotic process in the kidney because of the imbalance between profibrotic and antifibrotic molecules. The antifibrotic/anti-EndMT effects of AcSDKP were associated with restoration of the FGF receptor's levels and associated induction of microRNA let-7. Regard with this, microRNA let-7 family has been shown to protect kidney from fibrotic stimuli [17, 18].

## 5. Conclusion

In conclusion, AcSDKP is potentially a valuable endogenous antifibrotic molecule that inhibits the EndMT and restores the expression of the let-7 microRNA family through FGFR restoration at least in part. AcSDKP may therefore be useful for the clinical therapy for kidney fibrosis in diabetes.

## Supplementary Material

Figure 1: AcSDKP alone partially ameliorates kidney histology. Anti-fibrotic effects of AcSDKP have been well described. Therefore preliminary we tested anti-fibrotic effects of AcSDKP alone in small cohort. A, B. The kidney histology picture obtained from diabetic mice treated with AcSDKP alone. Representative A) PAS and B) MTS staining were shown. Scale bar: 50 µm. C-E. Morphometric analysis. The glomerular surface area (C), relative mesangial area (D), and relative area of fibrosis (E) were analyzed by the method described in the Materials and Methods section. F. Plasma concentration of AcSDKP in the diabetic mice treated with AcSDKP alone (n=2). These data are included in main Figure 2 C-D.
Figure 2: AcSDKP restored microRNA let-7 family. A. Total RNA, including the microRNA fraction, was harvested from the frozen kidney samples and microRNA array analysis was performed. n=2 in each group. The average value for each group is shown. In each data set, the relative level of expression compared to that of control mice is shown. B, C, D. qPCR analysis for the indicated members of the let-7 microRNA family. n=5 or 6 for each group. Diabetes is designated as DM.
