# Supplementary material for: N-acetyl-seryl-aspartyl-lysyl-proline Inhibits Diabetes-Associated Kidney Fibrosis and Endothelial-Mesenchymal Transition
Source: Biomed Res Int. 2014 Mar 24;2014:696475. doi: 10.1155/2014/696475 (PMC3982268; doi:10.1155/2014/696475)
Supplement: Supplementary file 1 — Figure 1: AcSDKP alone partially ameliorates kidney histology. Anti-fibrotic effects of AcSDKP have been well described. Therefore preliminary we tested anti-fibrotic effects of AcSDKP alone in small cohort. A, B. The kidney histology picture obtained from diabetic mice treated with AcSDKP alone. Representative A) PAS and B) MTS staining were shown. Scale bar: 50 µm. C-E. Morphometric analysis. The glomerular surface area (C), relative mesangial area (D), and relative area of fibrosis (E) were analyzed by the method described in the Materials and Methods section. F. Plasma concentration of AcSDKP in the diabetic mice treated with AcSDKP alone (n=2). These data are included in main Figure 2 C-D. Figure 2: AcSDKP restored microRNA let-7 family. A. Total RNA, including the microRNA fraction, was harvested from the frozen kidney samples and microRNA array analysis was performed. n=2 in each group. The average value for each group is shown. In each data set, the relative level of expression compared to that of control mice is shown. B, C, D. qPCR analysis for the indicated members of the let-7 microRNA family. n=5 or 6 for each group. Diabetes is designated as DM. [file 696475.f1.pdf]

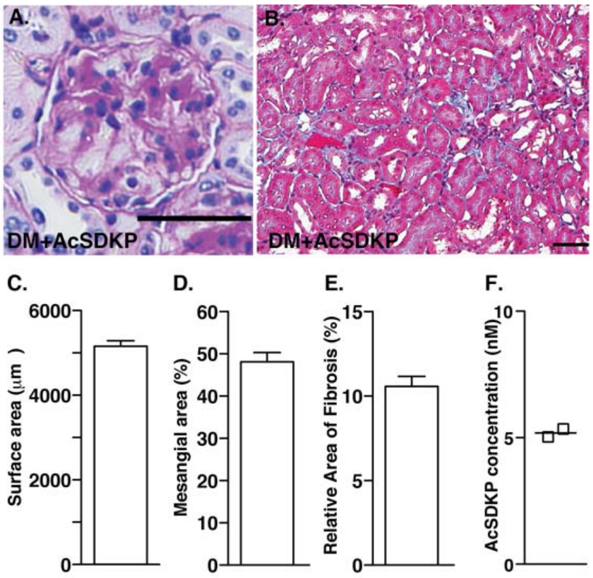

Supple. Figure 1: AcSDKP alone partially ameliorates kidney histology.
Anti-fibrotic effects of AcSDKP have been well described. Therefore preliminary we tested anti-fibrotic effects of AcSDKP alone in small cohort. **A, B.** The kidney histology picture obtained from diabetic mice treated with AcSDKP alone. Representative A) PAS and B) MTS staining were shown. Scale bar: 50 μm. **C-E**. Morphometric analysis. The glomerular surface area (**C**), relative mesangial area (**D**), and relative area of fibrosis (**E**) were analyzed by the method described in the Materials and Methods section. **F.** Plasma concentration of AcSDKP in the diabetic mice treated with AcSDKP alone (n=2). These data are included in main **Figure 2 C-D**.

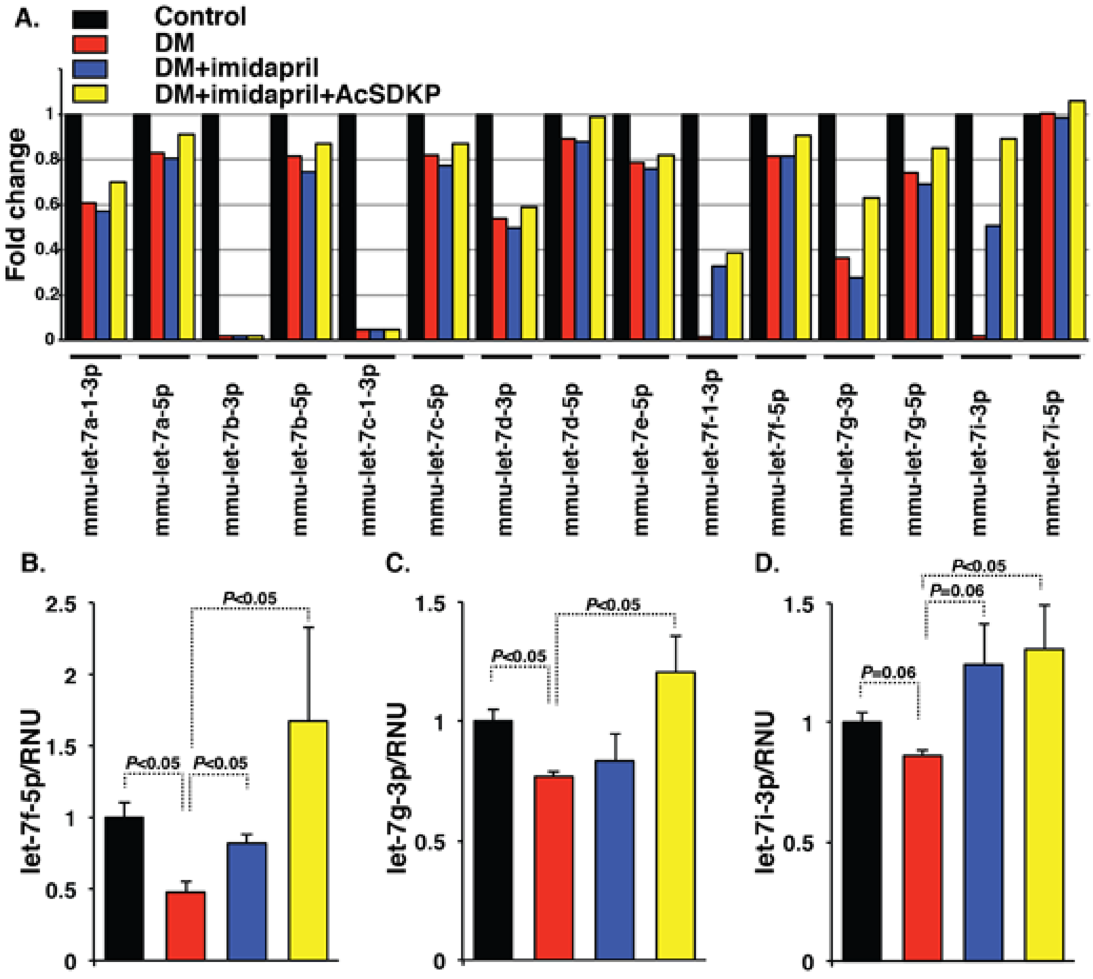

Supple. Figure 2: AcSDKP restored microRNA let-7 family. A. Total RNA, including the microRNA fraction, was harvested from the frozen kidney samples and microRNA array analysis was performed. n=2 in each group. The average value for each group is shown. In each data set, the relative level of expression compared to that of control mice is shown. **B, C, D**. qPCR analysis for the indicated members of the let-7 microRNA family. n=5 or 6 for each group. Diabetes is designated as DM.
